# Efficacy of Peer-Led Interventions to Reduce Unprotected Anal Intercourse among Men Who Have Sex with Men: A Meta-Analysis

**DOI:** 10.1371/journal.pone.0090788

**Published:** 2014-03-10

**Authors:** Shaodong Ye, Lu Yin, Rivet Amico, Jane Simoni, Sten Vermund, Yuhua Ruan, Yiming Shao, Han-Zhu Qian

**Affiliations:** 1 Vanderbilt Institute for Global Health, Vanderbilt University School of Medicine, Nashville, Tennessee, United States of America; 2 State Key Laboratory for Infectious Disease Prevention and Control, and National Center for AIDS/STD Control and Prevention, Chinese Center for Disease Control and Prevention, Beijing, China; 3 Center for Health, Intervention and Prevention, University of Connecticut, Storrs, Connecticut, United States of America; 4 Department of Psychology, University of Washington, Seattle, Washington, United States of America; 5 Departments of Pediatrics, Vanderbilt University School of Medicine, Nashville, Tennessee, United States of America; 6 Division of Epidemiology, Departments of Medicine, Vanderbilt University School of Medicine, Nashville, Tennessee, United States of America; Rollins School of Public Health, United States of America

## Abstract

**Objective:**

To conduct a systematic review and meta-analysis to evaluate the efficacy of peer-led interventions in reducing unprotected anal intercourse (UAI) among men who have sex with men (MSM).

**Methods:**

Randomized clinical trials (RCTs), quasi-experimental studies, pre- and post-intervention studies without control groups, and serial cross-sectional assessments involving peers delivering interventions among MSM and published as of February 2012 were identified by systematically searching 13 electronic databases and cross-referencing. Effect sizes (ES) were calculated as the changes of standardized mean difference (SMD) in UAI between groups or pre-post intervention.

**Results:**

A total of 22 studies met the eligibility criteria, including five RCTs, six quasi-experimental studies, six pre-and-post intervention studies, and five serial cross-sectional intervention studies. We used 15 individual studies including 17 interventions for overall ES calculation; peer-led interventions reduced UAI with any sexual partners in meta-analysis (mean ES: -0.27; 95% confidence interval [CI]: −0.41, −0.13; *P*<0.01). Subgroup analyses demonstrated a statistically significant reduction on UAI in quasi-experimental studies (mean ES: −0.30; 95% CI: −0.50, −0.09; *P* = 0.01) and serial cross-sectional intervention studies (mean ES: −0.33; 95% CI: −0.57, −0.09; *P* = 0.01), but non-significant reduction in RCTs (mean ES: −0.15; 95% CI: −0.36, 0.07; *P* = 0.18) or pre- and post-intervention studies (mean ES: −0.29; 95% CI: −0.69, 0.11; *P* = 0.15). Heterogeneity was large across these 15 studies (*I* ^2^ = 77.5%; *P*<0.01), largely due to pre-and-post intervention studies and serial cross-sectional intervention studies.

**Conclusions:**

Peer-led HIV prevention interventions reduced the overall UAI among MSM, but the efficacy varied by study design. More RCTs are needed to evaluate the effect of peer-led interventions while minimizing potential bias.

## Introduction

Men who have sex with men (MSM) continue to represent the largest number of new HIV infections in North America and other parts of the world [1], [2], [3], [4], [5], primarily due to practicing unprotected anal intercourse (UAI). Approximately 30–45% of MSM reported regular UAI [6], [7], [8]. Effective behavioral intervention strategies are needed to promote safer sex among MSM, and one approach is the use of peers to deliver HIV-prevention interventions [9], [10], [11], [12].

Peer-led HIV intervention typically involves members of a specific at-risk group to influence and support members of the same group to maintain healthy sexual behaviors, change risky sexual behaviors, and modify norms [13], [14]. Peer educators are thought to be more likely to influence the behaviors of their peers since they are seen as credible and less judgmental role models. Peer educators are also thought to have good access to hidden populations who may have limited interaction with more traditional health programs. In addition, they are also perceived to be less expensive in comparison to professional healthcare providers [15], [16].

In 1991, a peer-led intervention study among MSM was reported to successfully increase condom use and reduce the number of sexual partners [17]. Peers have been deployed to help MSM negotiate complex prevention, care, substance abuse, and social service systems [18]. Peer-led interventions largely emanate from the diffusion of innovation model [19], [20], [21], [22], [23], [24], [25], [26], [27], although other health behavioral models are also used [28], [29], [30], [31], [32], [33], [34], [35], [36], [37], [38], [39]. Even when the underlying theoretical model is the same, a wide diversity in quality and characteristics of implementation is inherent in this evidence base. Further, time is a critical consideration; interventions in the early 1990s were contextualized by limited treatment for HIV whereas those in the mid-2000s onward occur in the context of available antiretroviral therapies and new sexual cultures. However, even with heterogeneity in these factors, systematic reviews and selected meta-analysis can substantially contribute to the literature by estimating both the overall effects of peer-led interventions and dissecting potential differential effects on the basis of study- or population-related factors. A meta-analysis published in 2009 showed that peer education programs in developing countries were moderately effective at increasing HIV knowledge and increasing condom use, but had a nonsignificant impact on sexually transmitted infections (STIs) [40]. A systematic review published in 2011 suggested that more data are needed demonstrating an effect in the most rigorous study designs and with outcomes that are not potentially affected by respondent bias. We conducted an updated systematic review and meta-analysis of the peer intervention studies for reducing UAI among MSM.

## Methods

### Search strategy

A systematic literature search was performed to identify randomized clinical trials (RCTs), quasi-experimental studies, pre- and post-intervention studies without control groups, and serial cross-sectional intervention studies involving peers delivering interventions among MSM published as of February 29, 2012. The search was conducted in 13 electronic databases: AMED (Allied and Complementary Medicine Database, Ovid Technologies, Inc., New York), BIOSIS Previews (Biological Abstracts & Biological Abstracts/RRM, Thomson Scientific Technical Support, New York), British Nursing Index (Ovid Technologies, Inc., New York), EMBASE (Elsevier, Amsterdam, The Netherlands), EconLit (The American Economic Association, New York), ERIC (Education Resources Information Centre, Institute of Education Sciences of the U.S. Department of Education, Washington), Ovid Medline (Ovid Technologies, Inc., New York), PsycINFO (American Psychological Association, Washington), Scopus (Elsevier, Amsterdam, The Netherlands), Web of Science (Thomson Scientific Technical Support, New York), CNKI (Tongfang Knowledge Network Technology Co., Ltd., Beijing, China), CQVIP (Chongqing VIP Information Co., Ltd., Chongqing, China), and Wanfang Data (Chinese Ministry of Science & Technology, Beijing, China). Keywords used in the database search included: [(men who have sex with men) OR (MSM) OR (homosexual men) OR (gay men) OR (bisexual men) OR (transgender women) OR (money boy)] AND [(HIV) OR (AIDS) OR (sexually transmitted infections) OR (sexually transmitted diseases)] AND [(peer) OR (opinion leader)]. All publications were exported to an Endnote file (Endnote X4, Thomson Reuters, San Francisco, CA), and the duplicates were deleted.

### Study selection

Studies were selected if they met the following inclusion criteria: original RCTs, quasi-experimental intervention studies, pre- and post-intervention studies without control groups, or serial cross-sectional intervention studies of interventions among cohorts of MSM (or serial cross-sectional intervention studies); utilized MSM peers as intervention deliverers; reported UAI or condom use during anal sex before and after intervention between arms (RCTs and quasi-experimental studies), or only in intervention arm (pre- and post-intervention studies and serial cross-sectional intervention studies).

All abstracts were independently reviewed by two authors (SY and LY), and full texts were reviewed for determining eligibility if abstracts were incomplete. Manuscripts that met inclusion criteria were retained for full analysis. Any disagreements were resolved by further discussion involving an additional author (HZQ).

### Data extraction

For all eligible studies, two authors extracted the following information independently, using common abstraction forms: authors, publication year, study country, study design, description of interventions in study arms, training of peers, theoretical basis of intervention, sample sizes and retention rates in study arms, durations of follow-up, type (regular or casual) and HIV status (positive, negative, or unknown) of sex partners, position of anal sexual intercourse (insertive, receptive, or both), and proportions and mean frequencies of UAI. Disagreements were discussed until a consensus was reached.

### Rigor score

We assessed the rigor of the study design of each included study using an 8-point scale [40], plus an additional point for a samples size of ≥100. One point is awarded if a study met each of 9-item criteria; therefore, the total rigor score for each study may range from 0 to 9, with a larger value representing higher rigor of study design. If there were no data for one certain item, this item was scored as 0.5.

### Statistical methods

We used UAI as the outcome variable in this meta-analysis as this was the most common and HIV-relevant outcomes included in other studies. UAI was measured as continuous (e.g., frequency) or categorical variable (e.g., proportion) in the included studies. We adopted a conservative approach for calculating the proportion of UAI where the denominator was the number of total sample instead of the number of participants who reported UAI, as the latter may potentially overestimate the proportion of UAI. We calculated standard mean difference (SMD) in each study arm as a fraction of dividing the difference of two means at follow-up and baseline (or post- versus pre-intervention) by the pooled standard deviation (SD) of these two means. When studies reported dichotomous outcomes, odds ratios were transformed into SMD using Cox transformation [41]. We attempted to contact the authors if published articles did not provide the information needed to make the calculations.

We calculated the effect size (ES) of individual intervention as the difference of SMDs between study arms in RCTs and quasi-experimental studies, as well as in pre- and post-intervention studies and serial cross-sectional studies where we assumed a value of zero for SMD in the comparison arm [42]. Some studies had multiple intervention arms; in the case, we treated each intervention as an individual study while sharing the same comparison arm. Some studies had multiple measurements at different follow-up time points such that we used the last follow-up assessment for estimating the overall effect size. Each follow-up occasion was compared with the same baseline measurement in subgroup analyses by duration of follow-up. A negative value for ES indicates a greater reduction of UAI in the intervention arm relative to the comparison arm. Random effects models derived using the DerSimonian-Laird method [43], [44] were used to estimate overall effect sizes across studies. Random effects estimates allow for variation of true effects across studies [45]. As the study arms might not be comparable at baseline in quasi-experimental studies or even in RCTs, Becker's strategy was used to adjust for the differences [42].

Several planned subgroup analyses of studies were performed to examine effect sizes of any sexual partners, which were conducted by study design (e.g., RCTs, quasi-experimental studies, pre- and post-intervention studies, serial cross-sectional intervention studies); characteristics of risk assessment (e.g., recall period of UAI [>3 months, ≤3 months, or at last sex], position of anal intercourse [insertive or receptive], type and HIV-status of sexual partners [regular or casual, HIV negative or/and unknown status]); intervention characteristic (e.g., format of intervention delivery [group- or individual-based], theoretical base of intervention [diffusion of innovation or other theories]), as well as by other study characteristics including study country (US and Canada or China), number of study cities (one or multiple), venue of recruiting participants (establishment-based or other venues), sample size at baseline (≤300 or >300), publication year (prior to year 2000 or year 2000 or later), duration of follow-up (>12 months, 12 months, 7–11 months, 4-6 months, 3 months, or immediately after intervention), retention rate at the last follow-up (<80% or ≥80%), and rigor score (<6 or ≥6). We evaluated overall effect size based on 15 papers (17 interventions) as they reported UAI with any sexual partners; we included other 7 papers in subgroup analyses as they provided additional information such as UAI with regular or casual or with HIV negative or/and unknown status sexual partners [24], [25], [31], [32], [33], [37], [39].

Heterogeneity in overall efficacy and within specific subgroups was assessed by the *I* ^2^ statistic [46], and standardized deleted residuals analyses were performed to identify outliers. The funnel plot, Begg and Mazumdar rank correlation test, and Egger's test of the intercept were employed to assess publication bias [47].

Sensitivity analyses were conducted to determine the stability of intervention efficacy by evaluating whether the overall effect size was sensitive to inclusion of any given individual study. Studies excluded in iterative sensitivity analyses included those producing outliers identified by standardized deleted residuals analyses, involving two active intervention arms contrasted to the same control arm in the same study, involving only HIV-infected MSM participants, studies with low rigor score, or those with poor data reporting. All meta-analyses were performed in the R/S plus Software version 2.15.1.

## Results

### Results of literature search

**Figure 1:**
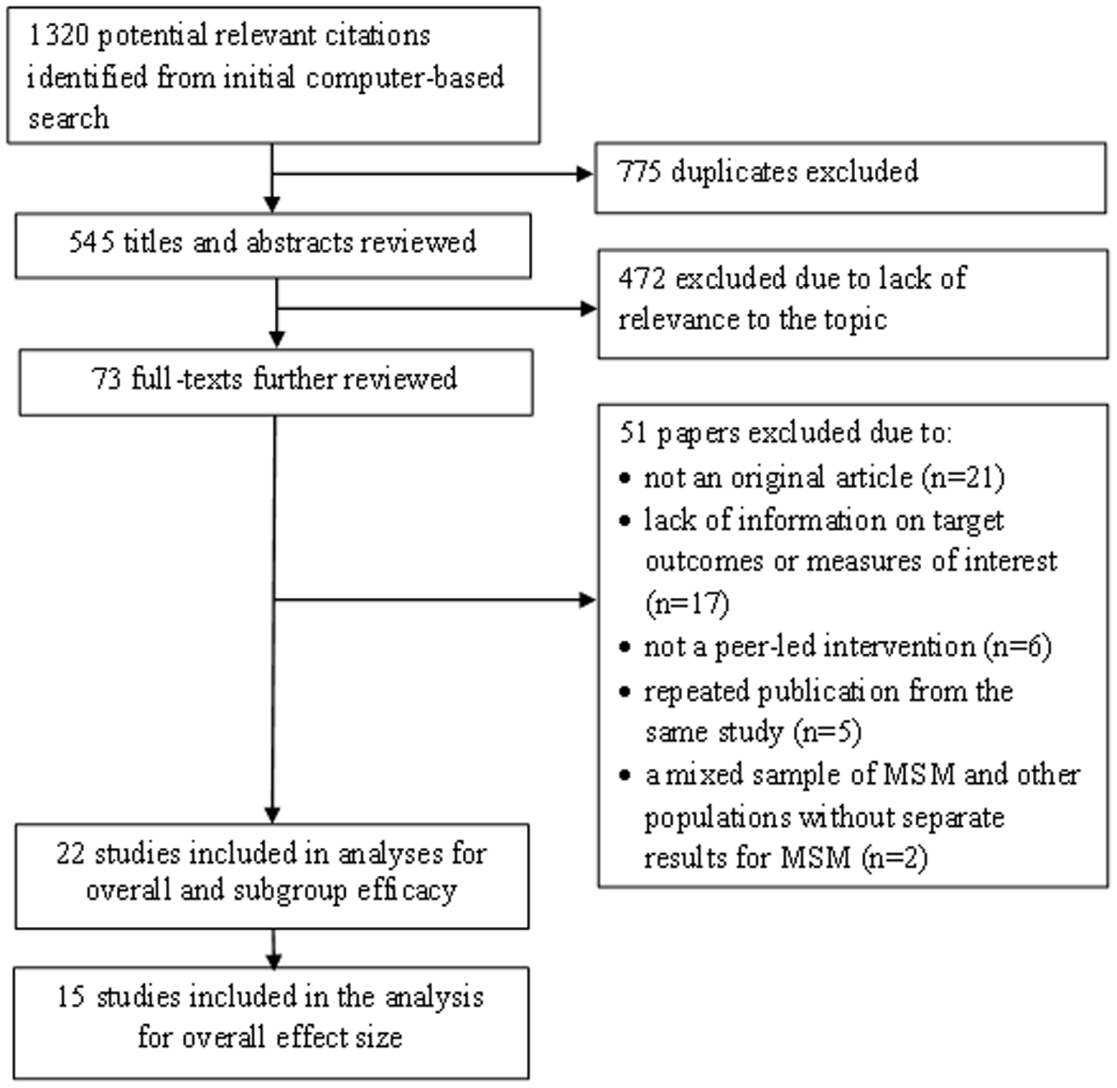
Flowchart of literature search and selection of studies.

The initial search of 13 individual electronic databases yielded 1,320 entries meeting our predefined inclusion criteria; 775 duplicates were identified and removed (Figure 1). Of the 545 remaining, 472 were excluded because they did not meet the inclusion criteria. Full text reviews of the remaining 73 papers led to further exclusion of 51 papers for the following reasons: not an original article (i.e., editorial, commentary, or review [n = 21]), lack of information on target outcomes or measures of interest (n = 17), not a peer-led intervention (n = 6), repeated publication from the same study (n = 5), and a mixed sample of MSM and other populations without separate outcomes for MSM (n = 2). These 51 studies are listed in Table S2. Thus, 22 publications met inclusion criteria for at least one of the planned analyses. Fifteen of the 22 studies had sufficient data for inclusion in the primary analysis of the overall efficacy on UAI with any sexual partners. Two of these 15 studies [28], [30] had two different interventions each, such that 15 publications including17 interventions were used for meta-analysis of the overall efficacy.

### Description of studies

Among the 22 studies included, 5 were RCTs [28], [30], [32], [35], [37]; 6 were quasi-experimental studies [17], [20], [21], [24], [25], [33]; 6 were pre- and post-intervention studies [23], [27], [29], [31], [34], [39]; and 5 serial cross-sectional intervention studies [19], [22], [26], [36], [38] (Table 1). The rigor score in these studies ranged from 2 to 9, with a mean of 4.8. Four studies had a score of 9 [28], [32], [35], [37] (Table 2).

**Table 1:** Characteristics of 22 peer-led HIV intervention studies among men who have sex with men.

			No. of participants at baseline and follow-ups		Study group					Follow-up (month)
Publication	Country (study year)	Study design*	Mean and range of participants' age, year)		Intervention			Comparison		
			Intervention	Comparison	Peer Training	Description	Theory			
Kelly et al. [17], 1991	USA (1989–1990)	Quasi	328278 (29, N/A)	331330 (29, N/A)	Three-stage, four weekly, 90-min group sessions for POL	POL conducted conversation with peers	Diffusion of innovation	No specified		6
Tudiver et al. [28], 1992	Canada (1990)	RCT	252201 (32, 14–72)	263212 (32, 14–72)	Not described	Single 180-min group session by POL	Face-to-face small group education technique	Wait-list control		3
			11188 (32, 14–72)			Four-weekly 120-min group session by POL				
Remafedi et al. [29], 1994	USA (1989–1991)	Pre-post	198139 (19, 12–21)	No	Not described	90 min single group session with peers	Cognitive and behavior adaptation	None		6
St Lawrence et al. [19], 1994	USA (N/A^2^)	SCIS	760614481 (N/A)	No	Not described	No described	Diffusion of innovation	None		36
Kegeles et al. [20], 1996	USA (N/A^2^)	Quasi	191103 (23, 18–29)	10988 (23, 18–29)	Not described	Peer outreach, single 180-min small group meeting with peers, publicity campaign	Modified diffusion of innovations	Wait-list control		12
Peterson et al. [30], 1996	USA (1989–1993)	RCT	1015564 (31, ≥18)	994750 (31, ≥18)	Single 5-hour training for POL	Single 180-min group session by POL	Social-cognitive intervention	Wait-list control		18
			1186671 (31, ≥18)			Three-weekly 180-min group sessions by POL				
Kelly et al. [21], 1997	USA (1991–1994)	Quasi	268199 (31, N/A)	174131 (31, N/A)	Five weekly, 120-min group sessions for POL	POL conducted conversation with peers	Diffusion of innovation	HIV-education materials		12
Miller et al. [22], 1998	USA (N/A)	SCIS	7201021 (31, 14–71)	No	2-weekly 3-session 120 min group sessions for POL	POL conducted conversation with peers	Diffusion of innovation	None		8
Kegeles et al. [23], 1999	USA (N/A)	Pre-Post	342194166 (23, 18–27)	No	Not described	Peer outreach, single 180-min small group meeting with peers, publicity campaign	Diffusion of innovation	None		12
Elford et al. [24], 2001	UK (1997–1999)	Quasi	472445 651612 (33, N/A)	532573 265108 (33, N/A)	Not described	POL conducted conversation with peers	Diffusion of innovation	Wait-list control		18
Flowers et al. [25], 2002	UK (1996–1999)	Quasi	12451442 (31, 15–40)	10311056 (31, 15–40)	2-day training for POL	POL conducted about 10-min conservation with peers	Diffusion of innovation	Routine sexual health services		36
Amirkhanian et al. [31], 2003	Russia & Bulgaria (2002)	Pre-Post	7772 (24, N/A)	No	Five-weekly 240-min training for POL	POL conducted conversation with peers	Multiple theory-based constructs	None		4
Wolitski et al. [32], 2005	USA (2000–2002)	RCT	413358 373 (N/A)	398335 354 (N/A)	Standard training	POL conducted six-weekly 180-min group sessions	Information-motivation-behavioral skills model	12- min single group session by local experts		6
Jones et al. [26], 2008	USA (2004–2005)	SCIS	295296317282 (23, 18–30)	No	Four 120-min training for POL	POL conducted conversation with peers	Diffusion of innovation	None		12
Zhu et al. [27], 2008	China (N/A)	Pre-post	218170 (24, 18–61)	No	Not described	POL conducted 4-session group conversion with peers	Modified diffusion of innovations	None		3
Zhang et al. [33], 2009	China (2007)	Quasi	200200 (N/A, 18–35+)	200200 (N/A, 18–35+)	4-session, 90-min training for POL	POL conducted conversion with peers	POL based integrated intervention model	Routine HIV prevention		6
He et al. [34], 2010	China (2006–2008)	Pre-post	360306 (23, 15–48)	No	Not described	POL conducted conversion with peers	No described	None		24
McKirnan, et al. [35], 2010	USA (2004–2006)	RCT	165131 (42, 18–50+)	148120 (42, 18–50+)	2400-min training for POL	POL conducted four 60–90-min individual counseling sessions	Motivation-interviewing and cognitive-behavioral techniques		Standard HIV primary care	12
Zhang et al. [36], 2010	China (2008–2009)	SCIS	315346 (27, N/A)	No	Not described	POL conducted 2–4 monthly conversion with peers	No described		None	12
Eaton et al. [37], 2011	USA (2009)	RCT	746460 (28, N/A)	756658 (30, N/A)	Not described	POL conducted single 20 min one-by-one session with peers	Conflict theory of decision-making		Standard HIV risk reduction counseling	3
Liang et al. [38], 2011	China (2009–2010)	SCIS	170252 (N/A)	No	Not described	POL conducted weekly conversion with peers	No described		None	12
Safren et al. [39], 2011	USA (N/A)	Pre-post	176138135137152 (43, 21–68)	No	Standard training	POL conducted 90-min four-session three-month conversion with peers	Peer-driven information-motivation-behavioral skill model		None	12

Notes: POLs = Popular opinion leaders; N/A = not available;

*Study design included randomized clinical trials (RCT), quasi-experimental studies (Quasi), pre- and post-intervention studies without control groups (Pre-post), and serial cross-sectional intervention studies (SCIS).

**Table 2:** Quality assessment of 22 included studies (rigor score^1^).

Publication	Cohort (a)	Control or comparison (b)	Pre/post intervention (c)	Random assignment (d)	Random selection for assessment (e)	Sample size>100 (f)	Follow-up≥80% (g)	Groups equivalent on Socio-demographics (h)	Groups equivalent at baseline on outcomes (i)	Final scores
Kelly et al. [17], 1991	1	1	1	0	0	1	1	1	1	7
Tudiver et al. [28], 1992	1	1	1	1	1	1	1	1	1	9
Remafedi et al. [29], 1994	1	0	1	0	0	1	0	0	0	3
St Lawrence et al. [19], 1994	0	0	1	0	0	1	0	0	0	2
Kegeles et al. [20], 1996	1	1	1	0	0	1	0	1	1	6
Peterson et al. [30], 1996	1	1	1	1	1	1	0	1	0	7
Kelly et al. [21], 1997	1	1	1	0	0	1	0	0.5	0	4.5
Miller et al. [22], 1998	0	0	1	0	0	1	0	0	0	2
Kegeles et al. [23], 1999	1	0	1	0	0	1	0	0	0	3
Elford et al. [24], 2001	1	1	1	0	0	1	0	0.5	1	5.5
Flowers et al. [25], 2002	0	1	1	0	0	1	0	1	1	5
Amirkhanian et al. [31], 2003	1	0	1	0	0	0	1	0	0	3
Wolitski et al. [32], 2005	1	1	1	1	1	1	1	1	1	9
Jones et al. [26], 2008	0	0	1	0	0	1	0	0	0	2
Zhu et al. [27], 2008	1	0	1	0	0	1	0	0	0	3
Zhang et al. [33], 2009	0	1	1	0	0	1	0	0	1	4
He et al. [34], 2010	1	0	1	0	0	1	1	0	0	4
McKirnan, et al. [35], 2010	1	1	1	1	1	1	1	1	1	9
Zhang et al. [36], 2010	0	0	1	0	0	1	0	0	0	2
Eaton et al. [37], 2011	1	1	1	1	1	1	1	1	1	9
Liang et al. [38], 2011	0	0	1	0	0	1	0	0	0	2
Safren et al. [39], 2011	1	0	1	0	0	1	1	0	0	4

1 One point score for meeting each of the following items (if data were not available in the articles for any item, 0.5 was recorded): (a) is a prospective cohort, (b) use a control or comparison group, (c) collect pre/post intervention data, (d) use random assignment of participants to study arms, (e) do random selection of subjects for assessment, (f) have comparison groups equivalent at baseline on outcome measures, sample size more than 100, (g) have a follow-up rate of 80% or more, (h) have comparison groups equivalent on socio-demographic measures, including age, education, race, employment, income, marital status and others [If more than half of variables were shown equivalent between groups, ‘1’ should be marked; otherwise “0”], and (i) have comparison groups equivalent at baseline on unprotected anal intercourse. If pre-post designs without comparison groups, “0” was recorded for item (h) and (i).

As indicated in Table 1, 13 of the 22 studies were conducted in the United States [17], [19], [20], [21], [22], [23], [26], [29], [30], [32], [35], [37], [39]; five in China [27], [33], [34], [36], [38]; two in United Kingdom [24], [25]; one in Canada [28]; and one in Russia and Bulgaria [31]. The sample size ranged from 77 to 2,276. Participants were recruited mostly by establishment-based sampling in venues frequented by MSM such as bars, clubs, and bathhouses. Other methods included peer-driven referrals, respondent-driven sampling, and advertisement-based approaches (e.g. websites, posters, radio). Duration of observation post initiation of intervention varied from 3 to 36 months post baseline assessment. For studies with control arms, the comparison condition was typically standard of care for HIV-prevention.

Nine studies developed the intervention based on Kelly's diffusion of innovation theory [17] with peer opinion leaders (POLs) [19], [20], [21], [22], [23], [24], [25], [26], [27], three used the cognitive behavioral theory [29], [30], [35], three used the information-motivation-behavioral skills model [32], [35], [39], and the remaining seven studies did not specify a theoretical background [28], [31], [33], [34], [36], [37], [38]. Some studies tailored behavioral theories to local cultures and study settings [34], [36], [38]. All interventions retained the basic element of peer-led intervention: a focus on changing cognition, behaviors, participatory learning, and social influence through peer educators (including POLs or peer counselors).

Nine studies did not provide information on selection and training of peer educators [19], [20], [23], [24], [28], [29], [36], [37], [38], while the other 13 did [17], [21], [22], [25], [26], [27], [30], [31], [32], [33], [34], [35], [39]. Peer educators often worked in group sessions and less frequently through one-on-one [35], [37]. They also distributed health information and condoms and assisted in recruiting or referring participants.

### Impact of peer-led interventions on UAI

The change in UAI by study arm, type of sexual partner, and position of anal sex (insertive or receptive) were described in Table S1. Most studies reported proportion of UAI, except two studies providing mean frequency of UAI [37], [39]. The effects of interventions varied considerably across individual studies.

**Figure 2:**
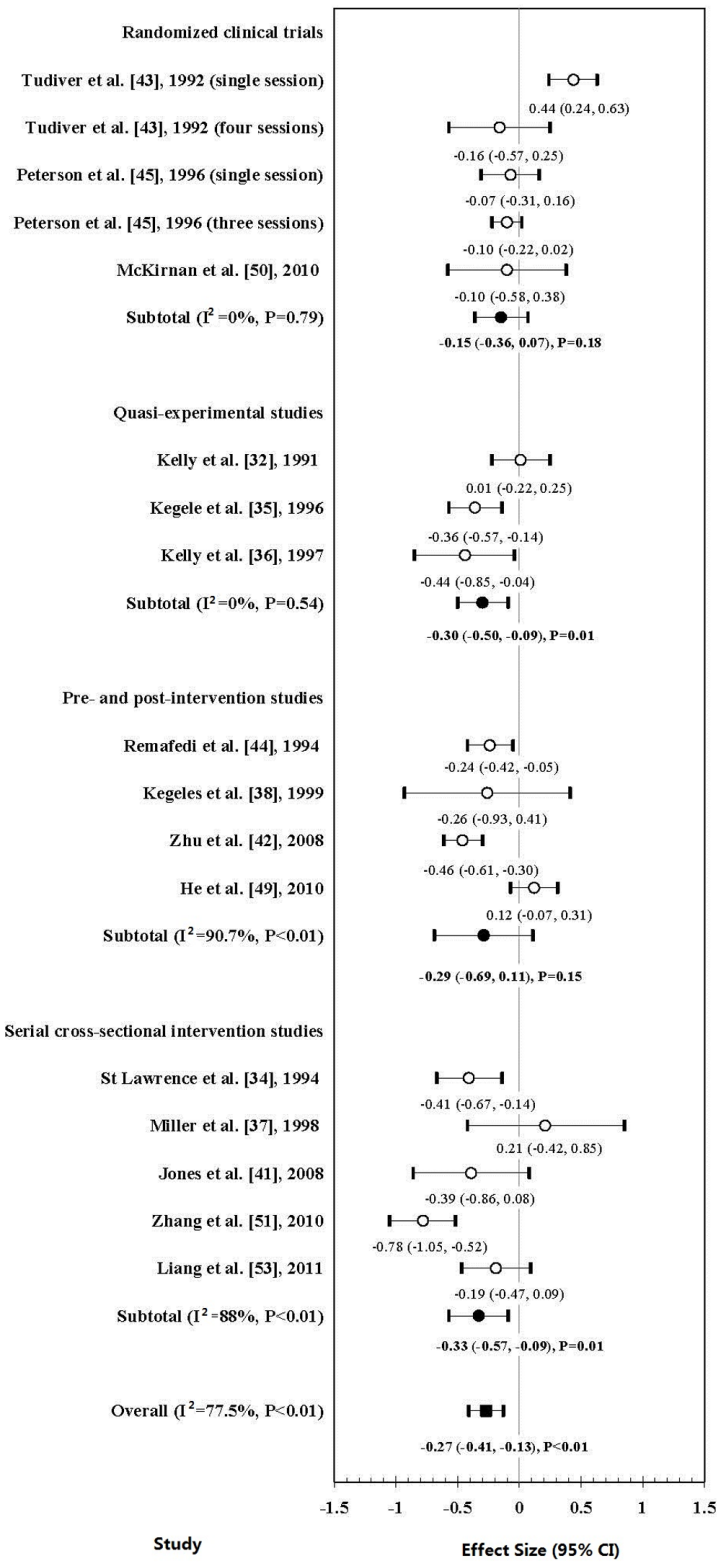
Forest plot of the effect sizes of peer-led interventions on change of unprotected anal intercourse among MSM in 15 studies (17 interventions).

**Figure 3:**
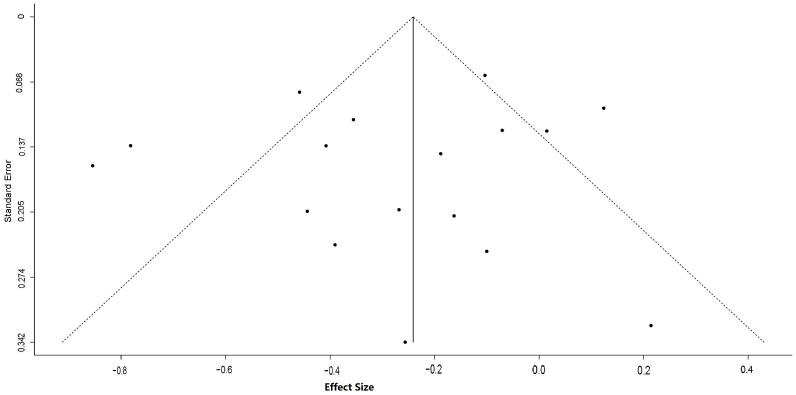
Funnel plot of 15 studies (17 interventions) for assessing publication bias. X-axis (horizontal) for the effect size or standard difference in means; y-axis (vertical) for the standard error of effect size.

Figure 2 shows the overall effect of the 15 studies which provided sufficient data to characterize SMD on UAI with any sexual partners. Fourteen studies demonstrated reduction in UAI, of which six reached statistical significance [19], [20], [21], [27], [29], [36]. The overall effect is statistically significant (mean ES: −0.27; 95% confidence interval [CI]: −0.41, −0.13; *P*<0.01), with significant heterogeneity across studies (*I* ^2^ = 77.5%; *P*<0.01). While quasi-experimental studies (mean ES: −0.33; 95% CI: −0.57, −0.09; *P* = 0.01; *I* ^2^ = 88%, *P*<0.01) and serial cross-sectional intervention studies (mean ES: −0.36; 95% CI, −0.56, −0.16; *P*<0.01; *I* ^2^ = 89%, *P*<0.01) showed statistically significant reduction in UAI, RCTs (mean ES: −0.15; 95% CI: −0.36, 0.07; *P* = 0.18; *I* ^2^ = 0%, *P* = 0.79) and pre- and post-intervention studies (mean ES: −0.29; 95% CI: −0.69, 0.11; *P* = 0.15; I^2^ = 90.7%, *P*<0.01) did not. No publication bias was detected based on the funnel plot (Figure 3) as well as Begg and Mazumdar rank correlation test and Egger's test (Kendall tau = 0; *P* = 1; Egger's t value = 0.14; *P* = 0.89).

Subgroup analyses and sensitive analyses showed peer-led interventions reduced UAI with casual sexual partners but not with regular sexual partners or with HIV negative or unknown status sexual partners (Table 3). Studies with a shorter duration of assessment produced statistically significant effects, whereas studies using a longer follow-up period (>12 months) did not. In bivariate analyses, studies of peer-led interventions from North America, conducted in multiple cities, reporting lower rates of retention, using establishment-based sampling, group-based intervention, or based on the diffusion of innovation theory showed statistically significant reduction in UAI.

**Table 3:** Subgroup meta-analyses and sensitivity analyses of UAI with any sexual partners.

Subgroup^1^	No. of studies	Combined ES (95% CI)	*P* value	Heterogeneity	
				*I* ^2^	*P* value
Characteristics of risk assessment					
Recall period of UAI (month)					
<3	9	−0.16 (−0.34, 0.02)	0.08	83.0%	<0.01
≥3	7	−0.25 (−0.53, 0.04)	0.10	84.7%	<0.01
Last sex	2	−0.07 (−0.72, 0.59)	0.84	93.7%	<0.01
Position of anal intercourse					
Insertive	3	−0.41 (−0.58, −0.24)	<0.01	37.3%	0.20
Receptive	3	−0.40 (−0.52, −0.27)	<0.01	0.0%	0.65
Type and HIV-status of sexual partners					
Regular	5	−0.28 (−0.58, 0.02)	0.07	73.7%	<0.01
Casual	6	−0.26 (−0.39, −0.12)	<0.01	0.0%	0.78
HIV negative/unknown status	4	−0.06 (−0.21, 0.08)	0.40	0.0%	0.42
Intervention characteristic					
Format of intervention delivery					
Individual-based	2	0.04 (−0.21, −0.30)	0.75	35.2%	0.21
Group-based	15	−0.32 (−0.46, −0.17)	0.01	74.5%	<0.01
Theoretical base of intervention					
Diffusion of innovation	8	−0.28 (−0.42, −0.15)	<0.01	62.1%	0.01
Other	9	−0.25 (−0.53, 0.04)	0.10	84.7%	<0.01
Study characteristic					
Study country					
America & Canada	13	−0.29 (−0.42, −0.15)	<0.01	65.2%	<0.01
China	4	−0.26 (−0.66, 0.15)	0.21	91.4%	<0.01
Number of study cities					
One	7	−0.18 (−0.40, 0.05)	0.12	81.3%	<0.01
Multiple	10	−0.36 (−0.51, −0.21)	<0.01	59.5%	0.01
Venue of recruiting participants					
Establishment-based	10	−0.31 (−0.48, −0.15)	<0.01	71.2%	<0.01
Other	7	−0.24 (−0.49, 0.02)	0.07	83.0%	<0.01
Sample sizes at baseline					
≤300	7	−0.32 (−0.57, −0.08)	0.01	73.6%	<0.01
>300	10	−0.24 (−0.42, −0.07)	0.01	80.3%	<0.01
Publication year					
Before year 2000	11	−0.29 (−0.45, −0.12)	<0.01	70.1%	<0.01
Year 2000 or later	6	−0.26 (−0.54, 0.02)	0.07	86.6%	<0.01
Durations of follow-up (month)					
Immediately after intervention	2	−0.24 (−0.35, −0.12)	<0.01	0.0%	0.70
3	3	−0.32 (−0.52, −0.12)	<0.01	0.0%	0.53
4–6	4	−0.39 (−0.70, −0.07)	0.02	79.0%	<0.01
7–11	2	−0.12 (−0.22, −0.01)	0.03	0.0%	0.68
12	10	−0.32 (−0.50, −0.14)	<0.01	69.0%	<0.01
>12	3	−0.11 (−0.07, 0.28)	0.24	0.0%	0.53
Retention rates at the last follow-up					
<80%	12	−0.34 (−0.51, −0.18)	<0.01	79.5%	<0.01
≥80%	5	−0.07 (−0.24, 0.10)	0.42	28.7%	0.23
Rigor score					
<6	10	−0.32 (−0.51, −0.13)	<0.01	86.8%	<0.01
≥6	7	−0.19 (−0.35, −0.03)	0.02	0.0%	0.86
Sensitivity analyses					
Tudiver et al. [28], 1992	15	−0.28 (−0.43, −0.13)	<0.01	80.2%	<0.01
Remafedi et al. [29], 1994	16	−0.24 (−0.37, −0.11)	<0.01	72.8%	<0.01
St Lawrence et al. [19], 1994	16	−0.26 (−0.40, −0.11)	<0.01	75.9%	<0.01
Peterson et al. [30], 1996	15	−0.29 (−0.44, −0.15)	<0.01	79.8%	<0.01
Miller et al. [22], 1998	16	−0.29 (−0.44, −0.13)	<0.01	76.8%	<0.01
Jones et al. [26], 2008	16	−0.27 (−0.42, −0.12)	<0.01	78.6%	<0.01
He et al. [34], 2010	16	−0.31 (−0.44, −0.17)	<0.01	72.8%	<0.01
McKirnan et al. [35], 2010	16	−0.28 (−0.42, −0.13)	<0.01	78.9%	<0.01
Zhang et al. [36], 2010	16	−0.24 (−0.37, −0.11)	<0.01	72.4%	<0.01
Liang et al. [38], 2011	16	−0.30 (−0.44, −0.15)	<0.01	77.4%	<0.01

1 A total of 22 studies were included in overall effect size estimation and in subgroup analyses. The sample sizes differ in each analysis due to data availability.

Notes: UAI = unprotected anal intercourse; ES = effect size; CI = confidence interval.

In standardized deleted residual analysis, two individual studies were identified as outliers [29], [36]. Further sensitivity analyses were used to evaluate the stability of the summary effect sizes. Iterative sensitivity analyses were conducted by excluding the studies which were identified as outliers [29], [36], used multiple intervention conditions [28], [30], involved HIV-infected MSM only [35], or had a low rigor score [19], [22], [26], [38] or poor data reporting [34]. Summary effect sizes do not change significantly by excluding any of above-described studies (Table 3).

## Discussion

Our systematic review of 22 studies and our meta-analysis of 17 interventions from 15 studies with qualifying UAI outcomes of peer-led interventions targeting MSM showed an overall beneficial effect on reducing UAI. Sensitivity analyses also showed reduction of UAI in all four types of study design, but the subtotal efficacy from RCTs (average SMD = -0.15) is not statistically significant and is smaller than those from other three study designs: serial cross-sectional intervention studies (SMD = −0.33), quasi-experimental studies (SMD = −0.30) and pre- and post-intervention studies (SMD = −0.29). High heterogeneity observed in these 15 included studies; and those employing serial cross-sectional or quasi-experimental design appeared to contribute significantly to the overall positive effect. Future studies should use more rigorous study design RCT to reduce potential bias. In this meta-analytic review, we did not assess the impact of pee-led interventions on disease rates such as HIV incidence [11], [40], [48], [49], [50], [51], [52].

Our meta-analysis found that follow-up assessments within 12 months showed a statistically significant relationship with reduction of UAI among MSM, whereas the few that examined longer-term (i.e., over a year) intervention effects did not have a significant average effect. Whether this reflects true dissipation of intervention effects or another factor is unknown. Given the scarcity of data for long-term outcomes, high quality peer-delivered intervention research that characterizes risk behavior beyond 12 months is needed.

UAI with casual sexual partners is known as a high risk factor for HIV acquisition. Recent research has indicated that higher levels of UAI may be associated with one's level of perceived familiarity with casual sexual partners [53]. Our meta-analysis showed that peer-led interventions significantly reduced UAI with casual sex partners, but did not reduce UAI with regular partners. Men may perceive regular partners less likely to transmit HIV, and therefore there is no need to take precautions in sex with stable partners.

Our stratified analysis by the format of delivering intervention found a 32% reduction of UAI for group-based interventions, but only a 4% (non-significant) increase for individual-based interventions. A previous meta-analysis found that individual-based interventions were more effective than group-based interventions to reduce UAI (51% vs. 34%), but it only included the studies published between 1988 and 2004 and focusing on HIV-infected persons [54]. Group-based intervention programs may be more cost-effective than individual-based interventions programs, and participants in group-based interventions have the opportunity of obtaining social support from multiple peers. These benefits of group-based versus individual-based interventions have been demonstrated in adult obesity and children's physical activity intervention programs [55], [56].

The diffusion of innovation model served as the theoretical basis for many peer interventions [19], [20], [21], [22], [23], [24], [25], [26], [27]. Our analysis showed that studies using this model appeared to be more successful in reducing UAI. Modified models of diffusion of innovation have been developed in UK [24], [25] and China [27]. Research on how to adapt this model to diverse cultures and communities is needed.

Our study is the first meta-analysis of the efficacy of peer-led interventions on UAI among MSM. Our analyses adjusted baseline data between study arms and combined continuous and categorical outcomes of UAI as reported in original studies. Therefore, our study has an advantage over previous reviews that failed to correct varying denominators [12], [18], [19], [22]. Our study also has limitations. We used UAI as the outcome of interest; self-report is subject to social desirability bias. We did not assess studies with biological outcomes including HIV infection. UAI may not reflect all beneficial effects of peer-led interventions [16]. We used all participating or successfully enrolled or followed-up MSM as the denominator for calculating UAI; this may underestimate effect estimates compared to using MSM who reported having anal sex as denominator. Pre- and post-intervention and serial cross-sectional intervention study designs represented about half of the included studies, which contributed a large portion of heterogeneity and may reduce the power of analysis.

In summary, our meta-analysis suggests that peer-led HIV prevention interventions have an overall impact on reducing UAI among MSM, but the efficacy varied by study design. Future peer-led intervention studies targeting MSM population should use RCT design for controlling the baseline difference between intervention and comparison arms, have a long follow-up period for assessing long-term effects of interventions, and use biological outcomes such as HIV seroconversion to reduce information bias.

## Supporting Information

Table S1 Efficacy of 22 peer-led interventions on unprotected anal intercourse (UAI) among men who have sex with men.
(DOCX)

Table S2 List of relevant studies excluded from this meta-analysis by category of reasons.
(DOCX)

Checklist S1 CONSORT checklist.
(DOC)
