# Supplementary material for: Efficacy of Peer-Led Interventions to Reduce Unprotected Anal Intercourse among Men Who Have Sex with Men: A Meta-Analysis
Source: PLoS One. 2014 Mar 10;9(3):e90788. doi: 10.1371/journal.pone.0090788 (PMC3948720; doi:10.1371/journal.pone.0090788)
Supplement: Table S1 — Efficacy of 22 peer-led interventions on unprotected anal intercourse (UAI) among men who have sex with men. (DOCX) [file pone.0090788.s001.docx]

**Table S1.** Efficacy of 22 peer-led interventions on unprotected anal intercourse (UAI)

among men who have sex with men

| **Publication** | **Proportion or mean frequency of UAI** | |
| --- | --- | --- |
|  | **Intervention arm** | **Comparison arm** |
| Kelly et al. [32], 1991 | Any anal intercourse 36.9🡪27.5^PM2,FM6^ | Any anal intercourse 40.4🡪37.5^PM2,FM6^ |
|  | Insertive anal intercourse 31.5🡪21.9^PM2,FM6^ | Insertive anal intercourse 31.5🡪31.9^PM2,FM6^ |
|  | Receptive anal intercourse 27.1🡪19.0^PM2,FM6^ | Receptive anal intercourse 30.9🡪27.2^PM2,FM6^ |
| Tudiver et al. [43], 1992 | Any anal intercourse | Any anal intercourse 22.9🡪18.3^PM3,FM3^ |
|  | Single group session 26.7🡪15.0^PM3,FM3^ |  |
|  | Serial group sessions 29.0🡪20.7^PM3,FM3^ |  |
| Remafedi et al. [44], 1994 | Current three partners 49🡪19^PM12,FM6^ | None |
| St Lawrence et al. [34], 1994 | Any anal intercourse 39.3🡪30.0^FM0^🡪23.3^PM2, FM12^ | None |
|  | Insertive anal intercourse32.5🡪23.8^PM0^🡪18.7^PM2, FM12^ |  |
|  | Receptive anal intercourse 28.8🡪20.3^PM0^🡪15.3^PM2,FM12^ |  |
| Kegeles et al. [35], 1996 | Any male partners 38.7🡪29.1^PM2,FM12^ | Any male partners 34.8🡪39.8^PM2,FM12^ |
|  | Causal male partners 19.4🡪10.7^PM2,FM12^ | Causal male partners 26.1🡪21.6^PM2,FM12^ |
|  | Regular male partners 41.7🡪20.4^PM2,FM12^ | Regular male partners 25.0🡪28.4^PM2,FM12^ |
| Peterson et al. [45], 1996 | Any anal intercourse | Any anal intercourse 32🡪23^FM12^🡪18^PM6, FM18^ |
|  | Triple group session 45🡪20^FM12^🡪20^PM6, FM18^ |  |
|  | Single group session 48🡪38^FM12^🡪38^PM6, FM18^ |  |
| Kelly et al. [36], 1997 | Any anal intercourse 32.1🡪20.3^PM2,FM12^ | Any anal intercourse 27.0🡪29.3^PM2,FM12^ |
| Miller et al. [37], 1998 | Any anal intercourse 37.1🡪33.2^PM2, FM8^ | None |
| Kegeles et al. [38], 1999 | Any male partners 37.7🡪30.4^FM0^🡪35.0^PM2, FM12^ | None |
|  | Causal male partners 18.8🡪13.4^FM0^🡪10.9^PM2, FM12^ |  |
|  | Regular male partners 21.9🡪19.6^FM0^🡪25.9^PM2, FM12^ |  |
| Elford et al. [39], | HIV status-unknown male partners | HIV status-unknown male partners |
| 2001 | 13🡪14^PM3,FM6^; 13🡪16^PM3,FM12^; 14🡪15^PM3,FM18^ | 15🡪11^PM3,FM6^; 7🡪14^PM3,FM12^; 15🡪12^PM3,FM18^ |
| Flowers et al. [40], 2002 | Causal male partners 11.6🡪10.9^PM12,FM36^ | Causal male partners 9.4🡪11.6^PM12,FM36^ |
| Amirkhanian et al. [46], 2003 | Causal male partners 32.5🡪22.2^PM3, FM4^; 19.5🡪11.1^PM0, FM4^ | None |
|  | Regular male partners 46.8🡪29.2^PM3, FM4^; 32.5🡪25.0^PM0, FM4^ |  |
| Wolitski et al. [47], 2005 | Any anal intercourse with HN/UP 35.1🡪26.5^FM3^🡪26.5^PM3,FM6^ | Any anal intercourse with HN/UP 35.5🡪31.3^FM3^🡪30.5^PM3, FM6^ |
|  | Insertive anal intercourse with HN/UP 18.9🡪14.2^FM3^🡪16.1^PM3,FM6^ | Insertive anal intercourse with HN/UP 21.9🡪17.6^FM3^🡪17.5^PM3, FM6^ |
|  | Receptive anal intercourse with HN/UP 27.8🡪21.2^FM3^🡪21.2^PM3,FM6^ | Receptive anal intercourse with HN/UP 25.7🡪26.0^FM3^🡪22.6^PM3, FM6^ |
| Jones et al. [41], 2008 | Any anal intercourse 40.3🡪33.4^FM4^🡪34.4^FM8^🡪27.3^PM2,FM12^ | None |
|  | Insertive anal intercourse 28.1🡪25.0^FM4^🡪25.9^FM8^🡪18.1^PM2,FM12^ |  |
|  | Receptive anal intercourse 31.2🡪24.0^FM4^🡪23.0^FM8^🡪17.4^PM2,FM12^ |  |
| Zhu et al. [42], 2008 | Any male partners 39.9🡪25.3^PM0,FM3^ | None |
|  | Causal male partners 33.5🡪27.1^PM0,FM3^ |  |
|  | Regular male partners 47.7🡪31.2^PM0,FM3^ |  |
| Zhang et al. [41], 2009 | Causal male partners 11.5🡪9.5^PM0,FM6^ | Causal male partners 11.0🡪19.5^PM0, FM6^ |
|  | Regular male partners 28.5🡪20.5^PM0,FM6^ | Regular male partners 29.0🡪22.5^PM0, FM6^ |
|  | Commercial sexual workers 6.5🡪1.5^PM0,FM6^ | Commercial sexual workers 11.0🡪7.5^PM0,FM6^ |
| He et al. [49], 2010 | Any anal intercourse 57.2🡪62.1^PM6,FM24^; 33.6🡪44.4^PM1,FM24^; 30.6🡪40.5^PM0,FM24^ | None |
| McKirnan, et al. | Any male partners 52.1🡪38^FM6^🡪39^PM6,FM12^ | Any male partners 43.2🡪44^FM6^🡪37^PM6,FM12^ |
| [50], 2010 | HN/UP 33.6🡪19^FM6^🡪21^PM6,FM12^ | HN/UP 25.0🡪24^FM6^🡪23^PM6,FM12^ |
| Zhang et al. [51], 2010 | Any anal intercourse 90.2🡪71.7^PM6,FM12^ | None |
| Eaton et al. [52], 2011 | HIV negative partners (mean/SE) 2.12/0.67^FM1^🡪1.03/0.39^PM1,FM3^ | HIV negative partner (mean/SE) 1.08/0.35^FM1^🡪1.14/0.47^PM1,FM3^ |
|  | HIV positive/unknown partners (mean/SE) 0.26/0.10^FM1^🡪0.30/0.14^PM1,FM3^ | HIV positive/unknown partners (mean/SE) 0.79/0.20^FM1^🡪0.44/0.15^PM1,FM3^ |
| Liang et al. [[53](#_ENREF_53)], 2011 | Any anal intercourse 49.4🡪50.0^PM12, FM12^; 30.0🡪34.9^PM0, FM12^ | No |
| Safren et al. [[54](#_ENREF_54)], 2011 | HN/UP (mean/SD) 2.57/10.77🡪3.60/21.28^FM3^🡪1.00/3.54^FM6^ 🡪2.23/9.52^FM9^🡪2.13/8.13^PM3, FM12^ | No |

Notes: HN/UP=HIV-negative or/and unknown sexual partners; SE=standard error; SD=standard deviation; PM0=at last sex; PM1-PM12: in the past 1 to 12 months; FM0: immediately after intervention; FM3-FM18: in the 3 to 18 follow-up months.
