# Supplementary material for: Efficacy of Peer-Led Interventions to Reduce Unprotected Anal Intercourse among Men Who Have Sex with Men: A Meta-Analysis
Source: PLoS One. 2014 Mar 10;9(3):e90788. doi: 10.1371/journal.pone.0090788 (PMC3948720; doi:10.1371/journal.pone.0090788)
Supplement: Table S2 — List of relevant studies excluded from this meta-analysis by category of reasons. (DOCX) [file pone.0090788.s002.docx]

**Table S2.** List of relevant studies excluded from this meta-analysis by category of reasons

*I.* *Not an original article but an editorial, comment or review*

1. Makuluwich GS. Programs to reduce HIV risk behaviors among gay men hinge on social networks. AIDS Patient Care STDS 1997;11(4):297-8.
2. Hart GJ. Peer education and community based HIV prevention for homosexual men: peer led, evidence based, or fashion driven? Sex Transm Infect 1998;74(2):87-9.
3. Wilson D, Cawthorne P. 'Face up to the truth': helping gay men in Vietnam protect themselves from AIDS. Int J STD AIDS 1999;10(1):63-6.
4. Elford J, Hart G, Sherr L, Williamson L, Bolding G. Peer led HIV prevention among homosexual men in Britain. Sex Transm Infect 2002;78(3):158-9.
5. Elford J, Sherr L, Bolding G, Serle F, Maguire M. Peer-led HIV prevention among gay men in London: process evaluation. AIDS Care 2002;14(3):351-60.
6. Hays RB, Rebchook GM, Kegeles SM. The Mpowerment Project: community-building with young gay and bisexual men to prevent HIV1. Am J Community Psychol 2003;31(3-4):301-12.
7. Elford J, Bolding G, Sherr L. Popular opinion leaders in London: a response to Kelly. AIDS Care 2004;16(2):151-8.
8. Hart GJ, Williamson LM, Flowers P. Good in parts: the Gay Men's Task Force in Glasgow--a response to Kelly. AIDS Care 2004;16(2):159-65.
9. Kelly JA. Popular opinion leaders and HIV prevention peer education: resolving discrepant findings, and implications for the development of effective community programmes. AIDS Care 2004;16(2):139-50.
10. Herbst JH, Sherba RT, Crepaz N, Deluca JB, Zohrabyan L, Stall RD, et al. A meta-analytic review of HIV behavioral interventions for reducing sexual risk behavior of men who have sex with men. J Acquir Immune Defic Syndr 2005;39(2):228-41.
11. Knauz RO, Safren SA, O'Cleirigh C, Capistrant BD, Driskell JR, Aguilar D, et al. Developing an HIV-prevention intervention for HIV-infected men who have sex with men in HIV care: project enhance. AIDS Behav 2007;11(5 Suppl):S117-26.
12. NIMH Collaborative HIV/STD Prevention Trial Group. Formative study conducted in five countries to adapt the community popular opinion leader intervention. AIDS 2007;21 (Suppl 2):S91-8.
13. Kim CR, Free C. Recent evaluations of the peer-led approach in adolescent sexual health education: a systematic review. Perspect Sex Reprod Health 2008;40(3):144-51.
14. Kim CR, Free C. Recent evaluations of the peer-led approach in adolescent sexual health education: a systematic review. Int Fam Plan Perspect 2008;34(2):89-96.
15. Medley A, Kennedy C, O'Reilly K, Sweat M. Effectiveness of peer education interventions for HIV prevention in developing countries: a systematic review and meta-analysis. AIDS Educ Prev 2009;21(3):181-206.
16. Neville S, Adams J. Condom use in men who have sex with men: a literature review. Contemp Nurse 2009;33(2):130-9.
17. Vergidis PI, Falagas ME. Meta-analyses on behavioral interventions to reduce the risk of transmission of HIV. Infect Dis Clin North Am 2009;23(2):309-14.
18. Malta M, Magnanini MM, Mello MB, Pascom AR, Linhares Y, Bastos FI. HIV prevalence among female sex workers, drug users and men who have sex with men in Brazil: a systematic review and meta-analysis. BMC Public Health 2010;10:317.
19. Chow EP, Wilson DP, Zhang L. What is the potential for bisexual men in China to act as a bridge of HIV transmission to the female population? Behavioural evidence from a systematic review and meta-analysis. BMC Infect Dis 2011;11:242.
20. Simoni JM, Nelson KM, Franks JC, Yard SS, Lehavot K. Are peer interventions for HIV efficacious? A systematic review. AIDS and Behavior 2011;15(8):1589-95.
21. Rajasingham R, Mimiaga MJ, White JM, Pinkston MM, Baden RP, Mitty JA. A systematic review of behavioral and treatment outcome studies among HIV-infected men who have sex with men who abuse crystal methamphetamine. AIDS Patient Care STDS 2012;26(1):36-52.

*II.* *Lack of information on target outcomes or measures of interest*

1. Valdiserri RO, Lyter DW, Kingsley LA, Leviton LC, Schofield JW, Huggins J, et al. The effect of group education on improving attitudes about AIDS risk reduction. N Y State J Med 1987;87(5):272-8.
2. Valdiserri RO, Lyter DW, Leviton LC, Callahan CM, Kingsley LA, Rinaldo CR. AIDS prevention in homosexual and bisexual men: results of a randomized trial evaluating two risk reduction interventions. AIDS 1989;3(1):21-6.
3. Leviton LC, Valdiserri RO, Lyter DW, Callahan CM, Kingsley LA, Huggins J, et al. Preventing HIV infection in gay and bisexual men: experimental evaluation of attitude change from two risk reduction interventions. AIDS Educ Prev 1990;2(2):95-108.
4. Shepherd J, Weare K, Turner G. Peer-led sexual health promotion with young gay and bisexual men--Results of the HAPEER project. Health Education 1997;97(6):204-212.
5. Pinkerton SD, Holtgrave DR, DiFranceisco WJ, Stevenson LY, Kelly JA. Cost-effectiveness of a community-level HIV risk reduction intervention. Am J Public Health 1998;88(8):1239-42.
6. Tao G, Remafedi G. Economic evaluation of an HIV prevention intervention for gay and bisexual male adolescents. J Acquir Immune Defic Syndr Hum Retrovirol 1998;17(1):83-90.
7. French R, Power R, Mitchell S. An evaluation of peer-led STD/HIV prevention work in a public sex environment. AIDS Care 2000;12(2):225-34.
8. Ziersch A, Gaffney J, Tomlinson DR. STI prevention and the male sex industry in London: evaluating a pilot peer education programme. Sex Transm Infect 2000;76(6):447-53.
9. Toro-Alfonso J, Varas-Diaz N, Andujar-Bello I. Evaluation of an HIV/AIDS prevention intervention targeting Latino gay men and men who have sex with men in Puerto Rico. AIDS Educ Prev 2002;14(6):445-56.
10. Gao Y, Wang S, Zhang S. Success in adopting particatory approach to fight HIV through behavior change among gay men. Xian Dai Yu Fang Yi Xue 2005;32(11):1512-5. [Chinese].
11. Somerville GG, Diaz S, Davis S, Coleman KD, Taveras S. Adapting the popular opinion leader intervention for Latino young migrant men who have sex with men. AIDS Educ Prev 2006;18(4 Suppl A):137-48.
12. Wu CX, Yu Q, Zhu CC, Xu SD, Dai H. Influence of the companion education on the change of AIDS knowledge and attitudes among the MSM. J Public Health Prev Med 2006;17(5):16-19. [Chinese].
13. Gao MY, Wang S. Participatory communication and HIV/AIDS prevention in a Chinese marginalized (MSM) population. AIDS Care 2007;19(6):799-810.
14. Lau JT, Lau M, Cheung A, Tsui HY. A randomized controlled study to evaluate the efficacy of an Internet-based intervention in reducing HIV risk behaviors among men who have sex with men in Hong Kong. AIDS Care 2008;20(7):820-8.
15. Velasquez MM, von Sternberg K, Johnson DH, Green C, Carbonari JP, Parsons JT. Reducing sexual risk behaviors and alcohol use among HIV-positive men who have sex with men: a randomized clinical trial. J Consult Clin Psychol 2009;77(4):657-67.
16. Carlos JA, Bingham TA, Stueve A, Lauby J, Ayala G, Millett GA, et al. The role of peer support on condom use among Black and Latino MSM in three urban areas. AIDS Educ Prev 2010;22(5):430-44.
17. Young SD, Konda K, Caceres C, Galea J, Sung-Jae L, Salazar X, et al. Effect of a community popular opinion leader HIV/STI intervention on stigma in urban, coastal Peru. AIDS Behav 2011;15(5):930-7.

*III. Not a peer-led intervention*

1. Rosser BR, Bockting WO, Rugg DL, Robinson BB, Ross MW, Bauer GR, et al. A randomized controlled intervention trial of a sexual health approach to long-term HIV risk reduction for men who have sex with men: effects of the intervention on unsafe sexual behavior. AIDS Educ Prev 2002;14(3 Suppl A):59-71.
2. Koblin B, Chesney M, Coates T. Effects of a behavioural intervention to reduce acquisition of HIV infection among men who have sex with men: the EXPLORE randomised controlled study. Lancet 2004;364(9428):41-50.
3. Golden MR, Gift TL, Brewer DD, Fleming M, Hogben M, St Lawrence JS, et al. Peer referral for HIV case-finding among men who have sex with men. AIDS 2006;20(15):1961-8.
4. Gutierrez JP, McPherson S, Fakoya A, Matheou A, Bertozzi SM. Community-based prevention leads to an increase in condom use and a reduction in sexually transmitted infections (STIs) among men who have sex with men (MSM) and female sex workers (FSW): the Frontiers Prevention Project (FPP) evaluation results. BMC Public Health 2010;10:497.
5. Outlaw AY, Naar-King S, Parsons JT, Green-Jones M, Janisse H, Secord E. Using motivational interviewing in HIV field outreach with young African American men who have sex with men: a randomized clinical trial. Am J Public Health 2010;100 Suppl 1:S146-51.
6. Rhodes SD, Hergenrather KC, Duncan J, Vissman AT, Miller C, Wilkin AM, et al. A pilot intervention utilizing Internet chat rooms to prevent HIV risk behaviors among men who have sex with men. Public Health Rep 2010;125 (Suppl 1):29-37.

*IV. Repeated publication from the same study*

1. Kelly JA, St Lawrence JS, Stevenson LY, Hauth AC, Kalichman SC, Diaz YE, et al. Community AIDS/HIV risk reduction: the effects of endorsements by popular people in three cities. Am J Public Health 1992;82(11):1483-9.
2. Kahn JG, Kegeles SM, Hays R, Beltzer N. Cost-effectiveness of the Mpowerment Project, a community-level intervention for young gay men. J Acquir Immune Defic Syndr 2001;27(5):482-91.
3. Williamson LM, Hart GJ, Flowers P, Frankis JS, Der GJ. The Gay Men's Task Force: the impact of peer education on the sexual health behaviour of homosexual men in Glasgow. Sex Transm Infect 2001;77(6):427-32.
4. Fernandez MI, Bowen GS, Gay CL, Mattson TR, Bital E, Kelly JA. HIV, sex, and social change: applying ESID principles to HIV prevention research. Am J Community Psychol 2003;32(3-4):333-44.
5. Zhang H, Wu Z, Zheng Y, Wang J, Zhu J, Xu J. A pilot intervention to increase condom use and HIV testing and counseling among men who have sex with men in Anhui, China. J Acquir Immune Defic Syndr 2010;53(Suppl 1):S88-92.

*V. A mixed sample of MSM and other populations without separate results for MSM*

1. Wright ER, Gonzalez C, Werner JN, Laughner ST, Wallace M. Indiana Youth Access Project: a model for responding to the HIV risk behaviors of gay, lesbian, and bisexual youth in the heartland. J Adolesc Health 1998;23(2 Suppl):83-95.
2. Kelly JA, Amirkhanian YA, Kabakchieva E, Vassileva S, Vassilev B, McAuliffe TL, et al. Prevention of HIV and sexually transmitted diseases in high risk social networks of young Roma (Gypsy) men in Bulgaria: randomised controlled trial. BMJ 2006;333(7578):1098.
